# A novel five-way translocation t(7;11;9;22;9)(q22;q13;q34;q11.2;q34) involving Ph chromosome in a patient of chronic myeloid leukemia: a case report

**DOI:** 10.1186/1755-8166-5-20

**Published:** 2012-05-01

**Authors:** Sho Yokota, Yuichi Nakamura, Masami Bessho

**Affiliations:** 1 Department of Hematology, Tachikawa Sougo Hospital, 1-16-15, Nishiki-cho, Tachikawa, Tokyo, 190-8578, Japan; 2 Department of Hematology, Saitama Medical University Hospital, 38 Moro-Hongo, Moroyama-machi, Iruma-gun, Saitama, 350-0495, Japan

**Keywords:** CML, Variant Ph translocation, Five-way translocation

## Abstract

About 5-10 % of chronic myelogenous leukemia (CML) patients show variant Philadelphia (Ph) translocations. The formation mechanisms and clinical significance of variant Ph translocations remain unclear. We report a CML case with a novel five-way complex translocation. Although the result of initial G-banding was 46,XY,t(7;11;9)(q22;q13;q34),t(9;22)(q34;q11.2), fluorescence *in situ* hybridization (FISH) demonstrated t(7;11;9;22;9)(q22;q13;q34;q11.2;q34) consisting of sequential rearrangements involving five chromosomes. The patient was successfully treated by imatinib and obtained a major molecular response. To our knowledge, this is the tenth CML case with a complicated Ph translocation involving five chromosomes and the third one treated by imatinib. Good response with imatinib therapy suggested that a single-event rearrangement was involved in the chromosomal changes.

## Background

Chronic myelogenous leukemia (CML) is a clonal myeloproliferative disorder of primitive hematopoietic stem cells. Most CML patients show a Philadelphia (Ph) chromosome with the characteristic t(9;22)(q34;q11.2) translocation. However, about 5-10 % of Ph positive patients with CML show variant translocations. The formation mechanisms and clinical significance of variant Ph translocations remain unclear.

We describe a CML case with a novel five-way chromosomal translocation t(7;11;9;22;9)(q22;q13;q34;q11.2;q34), who has been successfully treated by imatinib. To our knowledge, this is the tenth CML case with a complicated Ph translocation involving five chromosomes, and the third one treated by imatinib.

### Case presentation

The patient was 58-year-old Japanese male with no significant medical history. He was found to have increased white blood cell count (WBC) at a medical checkup at his workplace and referred to our hospital. The laboratory data on admission showed that his WBC was 19.1 × 10^9^/L, with a differential of 67.5 % neutrophils, 5.5 % myelocytes, 3.0 % metamyelocytes, 6.0 % basophils, 1.5 % eosinophils, 3.0 % monocytes, 13.5 % lymphocytes. Hemoglobin concentration of 13.0 g/dL was within a normal range and platelet count of 390 × 10^9^/L was slightly elevated. Neutrophil alkaline phosphatase (NAP) score was decreased to 79 (control score, 170–285). Bone marrow aspirate showed marked hypercellularity. Reverse-transcription polymerase chain reaction (RT-PCR) of RNA from his bone marrow cells amplified major *BCR/ABL* chimeric transcript (b3a2 type). He was diagnosed as having CML in the chronic phase, then received treatment with orally imatinib at daily of 400 mg. He obtained a complete cytogenetic response as well as a major molecular response (MMR), as *BCR/ABL* transcripts have not been detected by quantitative RT-PCR analysis after thirteen months treatment. The MMR status has been maintained for 44 months.

**Figure 1:**
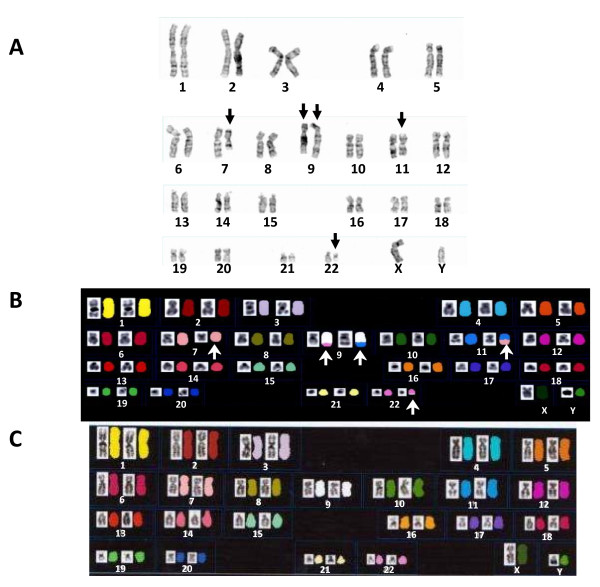
**(A) G-banded karyotype of the bone marrow cells.** The karyotype was initially decided as 46,XY,t(7;11;9)(q22;q13;q34),t(9;22)(q34;q11.2). Arrows indicate rearranged chromosomes. **(B)** Spectral karyotyping of the metaphase of the patient’s leukemic cells spread after spectrum-based classification. Chromosomes were assigned a pseudocolor according to the measured spectrum. Four derivative chromosomes, der(9)t(9;11)(q34;q13), der(9)t(9;22)(q34;q11), der(11)t(7;11)(q22;q13) and der(22)t(9;22)(q34;q11) and the truncated chromosome 7 were indicated by arrows. The grayscale images are reverse DAPI; the colored images, SKY. **(C)** Normal SKY image.

G-banding chromosomal analysis of the bone marrow cells presented 46,XY,t(7;11;9)(q22;q13;q34),t(9;22)(q34;q11.2) [20/20] (Figure 1A). After imatinib treatment, karyotype of the patient’s bone marrow cells showed 46,XY [20/20].

To confirm these cytogenetic aberrations, we performed Spectral karyotyping (SKY) analysis with a SkyPaint kit (Applied Spectral Imaging, Migdal Ha’Emek, Israel). As shown in Figure 1B, SKY confirmed four derivative chromosomes, der(9)t(9;11)(q34;q13), der(9)t(9;22)(q34;q11), der(11)t(7;11)(q22;q13), and der(22)t(9;22)(q34;q11). SKY could not visualize the small segment 9q34-9qter on the der(7)t(7;9)(q22;q34). The size of this segment was supposed to be smaller than a minimum genomic alteration that SKY could detect.

**Figure 2:**
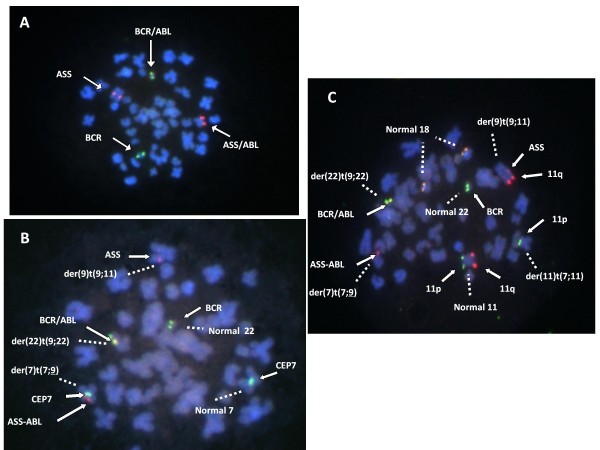
**FISH analyses with (A)*ABL*and*BCR*probes, (B) additional CEP7 probe and (C) additional 11p, 11q and chromosome 18 probes.** FISH probes and chromosomes of attention were indicated by arrows and dashed lines, respectively. **(A)** One yellow (red-green) signal, showing the *BCR/ABL* fusion, one *BCR* (green), one *ASS-ABL* (larger red), and one *ASS* (smaller red) signal were detected. **(B)** The segment (*ASS-ABL*, larger red signal) from the chromosome 9 was on der(7). The fusion *BCR/ABL* signal was on der (22), and the smaller red signal (*ASS*) was retained on the other chromosome 9. **(C)** The larger red signal, 11q, was on der(9) which retained the smaller red signal *ASS.*

For further characterization of these translocations, we next performed fluorescence *in situ* hybridization (FISH) analyses. In order to detect the *BCR/ABL* rearrangement and determine the 9q34 breakpoints, the LSI BCR/ABL ES Dual Color Translocation Probe Set (Vysis, Inc.) was used (assay 1). This probe set is a mixture of an *ABL* probe, which covers not only the *ABL* but also the centromeric located *ASS* gene (Spectrum Orange) and a *BCR* probe (Spectrum Green). In assay 1, one yellow (red-green) signal, showing the *BCR/ABL* fusion, one *BCR* (green), one *ASS-ABL* (larger red), and one *ASS* (smaller red) signal were detected (Figure 2A). This finding indicated that the two der(9) chromosomes had different 9q34 breakpoints, one within the *ABL*, generating the *BCR/ABL* fusion gene, the other within or centromeric to the *ASS*.

Additionally, we used two types of probe. One is the CEP7 (Vysis, Inc.) (Spectrum Green) that hybridizes to the centromere of chromosome 7 (assay 2). The other is the mixture consisted of 11p (Spectrum Green), 11q (Spectrum Orange), 18p (Spectrum Green and Spectrum Orange) and 18 centromeric (Spectrum Aqua) probes (Vysis, Inc. ) (assay 3). Assay 2 indicate that the *ASS-ABL* segment (larger red signal) from the chromosome 9 was on der(7) with CEP7 signal. The fusion *BCR/ABL* signal was on der(22), and the smaller red signal, *ASS*, was retained on the other chromosome 9 (Figure 2B). In assay 3, the larger red signal, 11q, was on one of der(9) which retained the smaller red signal *ASS* (Figure 2C), indicating that the 11q segment was translocated to a chromosome 9 with rearrangement within the *ABL*, but not to the other from which *ASS-ABL* was translocated to der(7).

**Figure 3:**
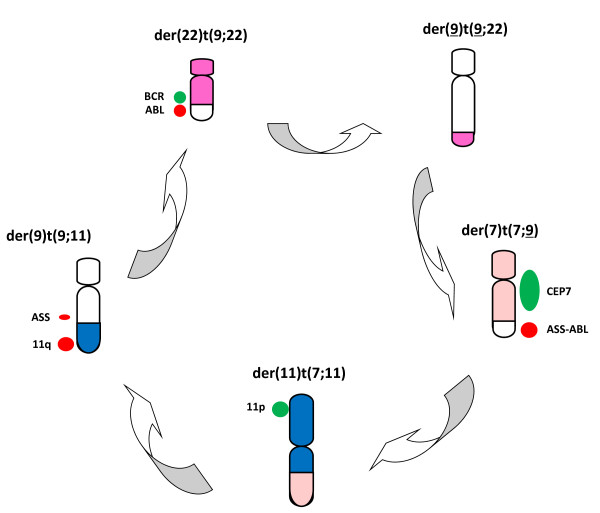
**Schema of the five-way translocation observed in this case.** Chromosomes were painted to the same colors as in SKY and FISH probes were shown by the side of chromosomes.

The results from G-banding, SKY and FISH analyses finally revised the karyotype as 46,XY,t(7;11;9;22;9)(q22;q13;q34;q11.2;q34) (Figure 3). We found nine other CML cases with five-way translocation, two of which were treated by imatinib [1-9] (Table 1). However, to our knowledge, this combination has not reported so far [10].

**Table 1:** Previously reported CML cases with five-way translocations and the present case

Case no.	Age/Sex	Karyotype of five-way translocation	Reference no.	Treatment	Outcome
1	10/Male	t(4;18;13;9;22)(q12;q11.2;q14;q34;q11.2)	[1]	BSF	Died 81months
2	23/Male	t(9;22;15;19;10)	[2]	Not reported	Not reported
3	68/Not reported	t(3;4;9;11;22)	[3]	Not reported	Died in benign phase
4	64/Female	t(9;22;21;11;inv ins(12)(q15p12p13)(q34;q11;q22;q13;q15)	[4]	BSF/VCR and PSL/ADM, CPA and MTX	Died 33 months
5	63/Male	t(9;22;10;12;1)(q34;q11.2;q22;p12;p36.1)	[5]	DNR,VCR,AND and PSL	Died 34 months
6	68/Female	t(9;22;15;13;17)(q34;q11;q26;q14;q11)	[6]	Chemotherapy	Not reported
7	Not reported/Male	t(2;9;16;22;22)(q32;q34;q21;q11;q11)	[7]	Not reported	Not reported
8	32/Male	t(4;12;7;9;22)(q33?;q24;p13;q34;q11)	[8]	GLI	41 months
9	45/Female	t(1;4;5;9;22)(q42;p14;q31;q34;q11.2)	[9]	HU/GLI	62 months
10	58/Male	t(7;11;9;22;9)(q22;q13;q34;q11.2;q34)	The present case	GLI	44 months

BSF:Busulphan, VCR:Vincristine, PSL: Prednisolone, ADM:Adriamycin, CPA:Cyclophosphamide, MTX:Methotrexate, DNR:Daunorubicin, GLI:Imatinib, HU:Hydroxyurea.

Two possible mechanisms have been postulated for formation of variant translocations. One is a single-event rearrangement via simultaneous breakage of several chromosomes followed by mismatched joining [11]. The other is a multi-step mechanism in which a classical Ph translocation is followed by further translocation events involving chromosomes 9 and 22 and other chromosomes [12]. These mechanisms may have prognostic importance in that a single genomic rearrangement may confer a similar prognosis to the classical Ph translocation, whereas a multi-step mechanism represents clonal evolutions associated with a worse prognosis [13].

Conflicting data were reported on clinical relevance of variant Ph translocation to tyrosine kinase inhibitor treatment [14-16] and its clinical significance has not been determined yet. Our case had achieved a MMR by imatinib therapy, suggesting that a single-event rearrangement was involved in the chromosomal change. However, careful follow-up will be needed, as complex translocations might be associated with a higher degree of genomic instability.

## Conclusions

We report a patient with CML presenting a complex five-way translocation, t(7;11;9;22;9)(q22;q13;q34;q11.2;q34). In our case, the initial finding on G-banding analysis suggested that an additional chromosomal aberration would occur independently from the Ph translocation. Chromosomal breaks occurred on both alleles of band 9q34 in the translocation, but only one of them was involved for the formation of *BCR/ABL* fusion. FISH method identified sequential rearrangements involving five chromosomes. Good response with imatinib therapy suggested that a single-event rearrangement was involved in the chromosomal changes.

### Consent

Written informed consent was obtained from the patient for publication of this case report and accompanying images. A copy of the written consent is available for review by the Editor-in-Chief of this Journal.

## Competing interests

The authors declare that they have no competing interests.

## Authors’ contributions

SY performed the cytogenetic studies in the present case and collected the data relative to this case report. SY, YN and MB did the molecular cytogenetic analysis and interpretation. SY and YN drafted the paper and all authors contributed to the finalizing of the manuscript. All authors read and approved the final manuscript.
